# Pennsylvania

**Published:** 1896-12

**Authors:** 


*Pennsylvania.* Some opposition to destroying tuberculous cows has arisen among the farmers of Montgomery County, adjacent to Philadelphia, where the milk is sent. As the Philadelphia press, in general, is clamoring for a purer milk-supply and rapidly educating the public, the agitation will work much good for the eradication of the disease in this section.

A Bucks County herd of 68 revealed 17 tuberculous animals and 2 suspicious ones in which the temperature was high, but not decisive.

Twenty-one head of thoroughbred cattle from Fayette County were recently destroyed at Pittsburg, and found to be much more seriously affected with tuberculosis than was indicated by the examination and tuberculin-tests applied.

